# Bis-Retinoid A2E Induces an Increase of Basic Fibroblast Growth Factor via Inhibition of Extracellular Signal-Regulated Kinases 1/2 Pathway in Retinal Pigment Epithelium Cells and Facilitates Phagocytosis

**DOI:** 10.3389/fnagi.2017.00043

**Published:** 2017-03-01

**Authors:** Delphine Balmer, Linda Bapst-Wicht, Aswin Pyakurel, Martine Emery, Natacha Nanchen, Christian G. Bochet, Raphael Roduit

**Affiliations:** ^1^Institute for Research in OphthalmologySion, Switzerland; ^2^Department of Ophthalmology, University of Lausanne, Jules-Gonin Eye Hospital, Fondation Asile des AveuglesLausanne, Switzerland; ^3^Department of Chemistry, University of FribourgFribourg, Switzerland

**Keywords:** retinal metabolism, A2E, age-related macular degeneration (ARMD), ARPE19, retinal pigment epithelium cells (RPE), fibroblast growth factor (FGF), mitogen-activated protein kinase (MAPK), extracellular signal-regulated kinase (ERK)

## Abstract

Age-related macular degeneration (ARMD) is the leading cause of vision loss in developed countries. Hallmarks of the disease are well known; indeed, this pathology is characterized by lipofuscin accumulation, is principally composed of lipid-containing residues of lysosomal digestion. The *N*-retinyl-*N*-retinylidene ethanolamine (A2E) retinoid which is thought to be a cytotoxic component for RPE is the best-characterized component of lipofuscin so far. Even if no direct correlation between A2E spatial distribution and lipofuscin fluorescence has been established in aged human RPE, modified forms or metabolites of A2E could be involved in ARMD pathology. Mitogen-activated protein kinase (MAPK) pathways have been involved in many pathologies, but not in ARMD. Therefore, we wanted to analyze the effects of A2E on MAPKs in polarized ARPE19 and isolated mouse RPE cells. We showed that long-term exposure of polarized ARPE19 cells to low A2E dose induces a strong decrease of the extracellular signal-regulated kinases' (ERK1/2) activity. In addition, we showed that A2E, via ERK1/2 decrease, induces a significant decrease of the retinal pigment epithelium-specific protein 65 kDa (RPE65) expression in ARPE19 cells and isolated mouse RPE. In the meantime, we showed that the decrease of ERK1/2 activity mediates an increase of basic fibroblast growth factor (bFGF) mRNA expression and secretion that induces an increase in phagocytosis via a paracrine effect. We suggest that the accumulation of deposits coming from outer segments (OS) could be explained by both an increase of bFGF-induced phagocytosis and by the decrease of clearance by A2E. The bFGF angiogenic protein may therefore be an attractive target to treat ARMD.

## Introduction

ARMD is the most significant cause of blindness in individuals over 50 years of age. This pathology is defined by the presence of drusens, hyper- and hypo-pigmentation of retinal pigment epithelium (RPE) cells, RPE and photoreceptor apoptosis and choroidal neovascularization (CNV). There are two subgroups of ARMD that are classically distinguished: the *dry* form (geographic atrophy) and the *wet* form (exudative). The *dry* form is characterized by the formation of drusen (extracellular debris and deposits) between the RPE cells and Bruch's membrane. This accumulation is toxic for RPE cells, which serve metabolic and supportive functions that are vital for retinal photoreceptors (Fine et al., 2000). The most important functions of RPE are (1) the regeneration of the bleached visual pigment (all-trans retinol to 11-cis retinal) allowing it to re-enter the visual cycle, (2) the maintenance of the interphotoreceptor matrix and Bruch's membrane, (3) the transport of oxygen, water, ions, and nourishment from the choroid vessels to the outer layers of the retina and (4) the phagocytic uptake and degradation of apical photoreceptor outer segments (OS) (for review see Lehmann et al., 2014). Drusen deposits are principally composed of lipofuscin resulting from free 11-cis-retinal that is continuously supplied to the rods for rhodopsin regeneration and outer segment renewal, and this altered material is trapped in the cell (Boyer et al., 2012). The *N*-retinyl-*N*-retinylidene ethanolamine (A2E) retinoid is the best-characterized constituent of lipofuscin and thought to be a cytotoxic component. Despite the fact that a recent study did not show any direct correlation between A2E and lipofuscin localization in human RPE (Ablonczy et al., 2013), it remains interesting to study the effects of this compound on RPE cells, because metabolites or modified forms of A2E could be involved in ARMD pathology. Moreover, A2E has a broad band absorption spectrum, with a peak in the blue range (~430 nm), therefore it is a potent photo-inducible generator of reactive oxygen species (ROS) which can damage proteins, lipids and DNA of RPE cells (Iriyama et al., 2008) leading to an impairment of RPE cell functions and to apoptosis (Suter et al., 2000). Importantly, A2E has been implicated in the activation of the complement system (Zhou et al., 2006) and in the induction of the vascular endothelial growth factor (VEGF) expression and secretion by RPE cells. This regulation occurs through the transactivation of the retinoic acid receptor (RAR) (Iriyama et al., 2008). As VEGF is thought to play a major role in the neovascularization which occurs in the later stage of ARMD, the accumulation of lipofuscin could be related to an increased risk of CNV (de Jong, 2006; Nowak, 2006). In addition, A2E inhibits retinoid isomerohydrolase activity by direct interaction with the retinal pigment epithelium-specific protein 65 kDa (RPE65), a key protein in the visual cycle (Moiseyev et al., 2010).

Fibroblast growth factor-2 (FGF2), also termed basic FGF (bFGF), is a member of the large FGF family. It fulfills numerous diverse functions, including stimulation of muscle cell growth, wound healing and tissue repair, and may play key roles in the nervous system, eye and skeleton (for review see Bikfalvi et al., 1997; Ornitz and Itoh, 2001). This angiogenic factor is expressed in almost all retinal cells and more interestingly in RPE cells (Bost et al., 1992). Three bFGF isoforms have been described in rodents (18, 21, and 23 kDa) and despite the lack of secretory signal peptide; the lower molecular weight isoform (18 kDa) is secreted, while higher molecular weight isoforms (21 and 23 kDa) are translocated to the nucleus. These isoforms have different subcellular localizations and functions (for review see Sørensen et al., 2006). The lower molecular weight isoform of bFGF (18 kDa) plays a role in cell growth and migration, in an autocrine manner. On the other hand, higher molecular weight isoforms of bFGF are principally involved in cell growth processes without affecting migration (for review see Bikfalvi et al., 1997). Secreted bFGF binds to heparin sulfate that first stabilizes the protein, besides creating a pool of angiogenic growth factors susceptible to be mobilized directly from the extracellular matrix to target cells (Powers et al., 2000). The release of bFGF occurs by exocytosis or by salting-out from damaged cells (Mignatti et al., 1992; Venuta et al., 2015). In addition to its angiogenic function, bFGF accomplishes autocrine and paracrine functions. In the retina, bFGF protects photoreceptors from light damage (Faktorovich et al., 1992), enhances the RPE phagocytic competence (McLaren and Inana, 1997; Sakuragi et al., 2001), induces retinal regeneration (Sakaguchi et al., 1997) and protects against photoreceptor degeneration (Faktorovich et al., 1990). On account of its presence in the interphotoreceptor matrix and RPE cells, it may protect the neural retina by regulating nitric oxide production (Goureau et al., 1993). In addition, bFGF has also been implicated in the up-regulation of VEGF, a key molecule in ARMD pathology (Seghezzi et al., 1998).

The human genome encodes 518 protein kinases, the so-called “kinome” (Manning et al., 2002). Our cells receive a wide range of stimuli and the activation of signal transduction cascades allows the cells to respond to all these divergent inputs in order to facilitate their adaptation to new environmental conditions. By phosphorylating substrate proteins, kinases modify the activity, the location and the affinities of up to 30% of all cellular proteins, and direct most cellular processes, particularly in signal transduction, and coordination of complex pathways. Recent reviews have highlighted the key roles of these protein kinases in numerous human diseases including inflammatory (rheumatoid arthritis, multiple sclerosis, asthma, inflammatory bowel disease, and psoriasis), neurodegenerative (Alzheimer's, Parkinson's and Huntington's diseases, stroke), metabolic (obesity, type 1, and 2 diabetes), and oncologic diseases (for review see Kim and Choi, 2010). The MAPKs (Mitogen-Activated Protein Kinases) are composed of three conserved kinases that proceed through a phosphorylation cascade creating a sequential activation pathway (Widmann et al., 1999). In mammals, three major MAPKs have been well described: (1) the ERK1/2/3 (Extracellular signal-Regulated Kinases 1-3; Davis, 1995), (2) the p38 kinases (P38 α/β/δ, and P38 g also named Erk6) (Enslen et al., 2000) and (3) the JNK1/2/3 (c-Jun N-terminal Kinases 1-3 Davis, 1994). Whereas, the ERKs are preferentially activated by growth factors and mitogens, the p38 kinases and JNKs primarily respond to a variety of stimuli collectively designated as “stress signals.” These include UV exposure, osmotic shocks and cytokine treatments (IL-1β, TNFα) and are associated with major changes in cell fate including growth arrest, apoptosis and activation of immune cells (for review see Pearson et al., 2001).

Within this study, we show that the polarization of ARPE19 cells (cultured on transwells vs. plastic plates) affects basal MAPK activity. Moreover, we first confirmed that A2E bis-retinoid is a toxic compound that induces ARPE19 cell death and describe for the first time that A2E modulates MAPK activities in polarized ARPE19 cells. While A2E rapidly activated the JNK pathway, the compound did not affect p38 and ERK1/2 pathways. Contrariwise, long-term exposure to A2E induced a significant decrease of ERK1/2, with no modification of p38 and JNK activities. A2E also decreased ERK1/2 activity in isolated mouse RPE, and in addition down-regulated the protein expression of RPE65. A2E drastically increased bFGF mRNA expression through the inhibition of ERK1/2, and seemed to promote bFGF secretion. Interestingly, bFGF was able to down-regulate ERK1/2 activity in polarized ARPE19 cells and promoted phagocytosis. We point out, for the first time, that A2E induces bFGF, an angiogenic factor that could play an important role in ARMD pathology. Therefore, bFGF could be, as well as VEGF, another interesting target in order to treat ARMD.

## Materials and methods

### Mouse line

This study adhered to the Association for Research in Vision and Ophthalmology (ARVO) statement for the use of animals in ophthalmic and vision research and was approved by the Veterinary service of the State of Valais, Switzerland (permit ID: VS22). Animals were kept in a 12-h light/12-h dark cycle with unlimited access to food and water. Mice were killed by cervical dislocation followed by exsanguination just after eye enucleation and retina isolation.

### A2E synthesis

A2E was synthesized following the protocol proposed by Parish et al. (1998), briefly all-trans-retinal (100 mg, 0.352 mmol, 2.0 equiv) was added to a solution of ethanolamine (9.5 mg, 0.155 mmol, 0.9 equiv) and acetic acid (9.3 ml, 155 mmol, 0.9 equiv) in 3.0 ml of ethanol. The mixture was stirred at room temperature in the dark for 44 h. The deep red solution was directly chromatographed without evaporation on a silica gel column (25 mm^*^20 cm), first eluting with 300 ml of CH_2_Cl_2_/MeOH (95:5 v/v), then with 300 ml of CH_2_Cl_2_/MeOH/CF_3_COOH (90:10:0.001 v/v). A2E was isolated from the dark orange fractions eluting immediately after the solvent change. The last fractions contained impurities and were discarded. The pure fractions (checked by LC-MS) contained 76.5 mg of A2E (0.108 mmol, 31%). 1H-NMR (CD_3_OD) was identical to published values (Parish et al., 1998). A2E was aliquoted, lyophilized and stored at −20°C in the dark. Prior to each experiment, A2E was reconstituted in ethanol (EtOH) and concentration assessed by near-UV (430 nm) aborption value as described by Parish et al. (1998).

### Isolated primary RPE and ARPE19 culture conditions

Primary RPE cells were obtained from isolated Retinas of 12 day-old C57BL/6 mice as previously described (Bonilha et al., 1999; Nandrot et al., 2004); briefly the eyes were dissected and placed directly in calcium- and magnesium-free Hank's buffered salt solution (HBSS) (Gibco, 14175-053). The cornea, lens, iris, and vitreous body were removed and eyes were treated with 1 mg/ml hyaluronidase (Sigma, H3506 451 U/mg) for 45 min at 37°C, in HBSS. Then, the eyecups were transferred into HBSS and the retina removed. Eyecups with exposed RPE were incubated for 45 min at 37°C in 2 mg/ml trypsin (Gibco, 27250-018) in HBSS containing calcium and magnesium (Gibco, 14065-049). Following digestion, they were transferred into calcium- and magnesium-free HBSS and patches of RPE were peeled off manually from the Bruch's membrane. These RPE sheets were removed from the underlying choroid with dissecting forceps and needles, then carefully collected and stored in a very small volume of HBSS. Afterwards, RPE sheets were incubated for 1 min at 37°C with trypsin/EDTA 0.25% (Sigma, T4049). Then, the cells were centrifuged at 1′000 rpm during 5 min, supernatant was discarded and cells resuspended in DMEM F12 medium (Gibco, 31330-095) supplemented with 10% heat-inactivated fetal bovine serum (FBS) (Lonza, #DE14-801F) and 1% penicillin/streptomycin (Gibco, 10378-016); and then plated on 12- or 6-well plates (Milian, TP-92412 or -06) for 5 days at 37°C before any treatment. Six eyes were needed per well of 12-well plates.

ARPE19 and isolated RPE cells were cultured on transwell filters (Corning® Transwell® polyester; Sigma, CLS3450-24EA; Dunn et al., 1996; Kannan et al., 2006), treated for 60 min at RT with 1% laminin (Sigma, L2020) in DMEM F12 without serum. Cells were seeded at a density of 500′000 cells/transwell and cultured for 3 days in DMEM F12 supplemented with 10% FBS and 1% penicillin/streptomycin (Gibco). Afterwards cells were cultured 4 weeks before treatment, in DMEM F12 supplemented with 1% FBS. ARPE19 cells were also cultured on plastic wells to confluence in DMEM F12 supplemented with 10% heat inactivated FBS for 1 month as previously described (Roduit and Schorderet, 2008). Isolated mouse RPE or ARPE19 cells were cultured in presence or absence of 5 μM A2E (protected from light), 200 ng/ml bFGF (Sigma, F0291) and 10 μM U0126 (Sigma, U120-1 mg) ERK1/2 inhibitor, for different periods of time prior mRNA or protein extraction for analysis. In major experiments, ARPE19 were cultured for 72 h in presence of A2E, then the medium was changed and cells further cultured for another 72 h period without A2E. For Cycloheximide (Santa Cruz Biotechnology, sc-3508) and Actinomycin (Sigma, A5156-1VL) experiments on bFGF mRNA expression, ARPE19 were prepared on filters as described above and then cultured for 48 h in absence or in presence of 5 μM A2E, with or without 10 μg/ml Cycloheximide or 5 μg/ml Actinomycin.

Concerning phagocytosis analysis, ARPE19 cells were grown to confluence on 0.1% gelatin-coated glass coverslips in DMEM F12 supplemented with 10% FBS. Then, the cells were supplemented with various additives to challenge their phagocytosis ability. A2E treatment was performed as described above, and prior phagocytosis assay, 5 μM U0126 was added for 24 h at 37°C, where cells were protected from light and 200 ng/ml bFGF was added into the media for 24 h at 37°C. Finally Merocyanine 540 (MC540) [Sigma, 323756] was used at a concentration of 4.5 μM in serum-free cell culture medium for 1 h at 37°C, then washed 3 times with PBS and irradiated for 30 min with a green range light (Philips TLD 18W/17). After irradiation, the cells were incubated for 24 h with fresh medium at 37°C.

### Western blot analysis and immunostaining

Thirty μg of total proteins were separated on acrylamide gels (10–12%) and electrically transferred onto PVDF filters. The membranes were then incubated with the following antibodies from Cell Signaling: anti-phospho-JNK (#4668), anti-phospho-p44/42 MAPK (Erk1/2) (4377S), anti-phospho-p38 (9211S) and anti-p38 (9212); from Abcam: anti-Erk2 (ab7948), anti-type 1 collagen (ab34710) and anti-RPE65 (ab13826); from Sigma: anti-bFGF (SAB3300013) and anti-alpha-tubulin (T5168); from Santa Cruz Biotechnology: anti-JNK (sc-571). We used AlexaFluor® 594 as secondary antibodies, either goat anti-mouse IgG (H+L) (LubioScience GmbH, A-11005) or goat anti-rabbit IgG (LubioScience GmbH, A11012) depending on the primary antibody used. All western blot analyses were performed under denaturing conditions, except for type 1 collagen detection where the native condition was kept, using tris-glycine migration buffer without SDS and commercial non-denaturing precast gel (BioRad, M062211A1). A protein ladder (Li-COR, 928-40000) was used to confirm the correct size of the proteins of interest.

Immunostaining for ZO-1 was done using anti-ZO-1 from Invitrogen (617300). ARPE19 cells were cultured as described above and then fixed with 4% paraformaldehyde (Acros Orga, 41678-0010) for 1 h at RT. After three successive washes of 10 min with PBS, the cells were permeabilized with 0.1% Triton X100 for 30 min at RT, and then incubated in blocking buffer (5% NGS (Sigma, G9023) for 1 h at RT, prior their incubation with anti-ZO-1 O/N at 4°C. An AlexaFluor® 594 goat anti-rabbit IgG was chosen as secondary antibody. Immunostaining for bFGF was performed without the permeabilization step in order to detect external bFGF; briefly the cells were cultured and treated as described above, fixed with 2% paraformaldehyde at the end of the experiment and incubated O/N at 4°C with anti-bFGF antibody (Sigma, F3393), and AlexaFluor® 594 goat anti-rabbit IgG was used as secondary antibody. Fluorescence was visualized under microscope (Leica) using appropriate filters.

### Cell death analysis

ARPE19 cell apoptosis was assessed after various periods of time (5, 72, and 72 + 72 h recovery) by staining with propidium iodide (Sigma, 81845-25 MG) and Hoechst 33342 (Sigma, B2261-25 MG). Apoptotic cells exhibited a condensed nuclear chromatin or a fragmented nuclear membrane when visualized with Hoechst 33,342. Necrotic cells were stained with propidium iodide at 1/2'000 dilution for 5 min at 37°C. Then the fluorescence was analyzed under microscope using appropriate filters.

### RT-PCR analysis

mRNA was extracted using Trizol solution (LucernaChem, Tri Reagent, TR-118-200) and then 800 ng of mRNA in a 20 μl reaction were used for cDNA synthesis using oligo (dT)18 according to the manufacturer's procedure (Roche Applied Science, First strand cDNA synthesis kit for RT-PCR #04897030001). The equivalent of 2–20 ng original total RNA was used for quantitative PCR amplification using the 2 x brilliant SYBR Green QPCR Master Mix (Agilent Technologies, #600548) and 0.5 mM forward and reverse primer pairs, designed to span an intron of the target gene. RPE65 was detected using forward primer (5′-CCCTTTTGCACAAGTTTGAC-3′) and reverse primer (5′-AAAGCACAGGTGCCAAATTC-3′), bFGF was detected using forward primer (5′-AGCGGCTGTACTGCAAAAAC-3′) and reverse primer (5′-AGCCAGGTAACGGTTAGCAC-3′). Normalization was performed with the GAPDH expression detected using forward primer (5′-ATGCCTCCTGCACCACCAAC-3′) and reverse primer (5′-CGCCTGCTTCACCACCTTCT-3′). Triplicate Real-time PCR was performed in the Light Cycler 480 from Roche, using the following cycling conditions: 1 cycle of denaturation at 95°C for 10 min, followed by 50 cycles of denaturation at 95°C for 10 s, annealing at 57°C for 45 s without any extension for all qPCR reactions.

### Photoreceptor outer segment (POS) preparation and fluosphere opsonization

POS were prepared from freshly slaughtered cow eyes following a protocol adapted from a previous study (Klettner et al., 2011). Briefly, 12 retinas needed 10 ml homogenizer buffer (34% sucrose, 65 mM NaCl, 2 mM MgCl_2_) and were vortexed for 2 min. The solution was then centrifuged 4 min at 3′800 rpm at room temperature, allowing cell bodies to be pelleted and POS to remain in solution due to the osmolarity of the buffer. The supernatant was diluted in 2 volumes of HEPES 10 mM, gently mixed and centrifuged again. The supernatant was discarded and the pellet, constituted of POS, was resuspended in 5 ml of 10 mM HEPES and homogenized 30 times through a 23 gauge canula. The protein concentration was assessed by the Micro BCA protein stain assay (Perbio Science Switzerland SA, 23235).

Yellow-green fluorescent FluoSpheres® beads (Molecular Probes, F-8853) of 2.0 μm diameter were opsonized by POS. FluoSpheres were prepared with a ratio of 0.5 mg of POS per 43.3 million beads (10 μl of commercial solution), mixed together for 1 h on a rotary wheel at 4°C in the dark. Coated beads were separated by centrifugation at 6,600 rpm for 20 min at 4°C. Then the pellet was washed twice, using sterile 0.9% NaCl solution, and resuspended in 100 μl of this latter solution. POS-coated Fluospheres were used for phagocytosis assay just after preparation.

### Fluosphere and POS phagocytosis assay

Twenty million beads of FluoSpheres alone or coated with POS were added per well in 12-well plate cultures (Milian SA, TP-92412) of ARPE19 cells treated with various compounds (see cell culture conditions section). After 16 h of incubation, the cells were washed 6 times with PBS to remove all unabsorbed FluoSpheres. Nuclei were stained with Hoechst dye (Sigma, B2261-25 MG, 1/2000) in normal medium during 5 min at 37°C. After 3 more washes with PBS, the cells were fixed using 4% paraformaldehyde (Acros Orga, 41678-0010) solution for 30 min at room temperature and then washed another 3 times with PBS and finally mounted on glass slides with one drop of citifluor (Citifluor Ltd, AF1). Finally, measurement of phagocytosis rate was done by microscopy analysis, the ratio between the area covered by Fluospheres and the number of cell nuclei was calculated with the Image J program for each picture (four squares per condition).

### Statistical analysis

All results were expressed as means ± SEM of the indicated number of experiments. Data were statistically analyzed using Prism 6.0 software. Comparison of two groups was done by using *T*-test analysis. For more than 2 groups, we first tested each group of data for distribution normality using Shapiro-Wilk tests. In case of normal distribution, we used a Welch's ANOVA test (one-way ANOVA with unequal variances) followed by a *post-hoc* Tukey-Kramer test to compare the different treatments. When the distribution was not normal, we used a Kruskal-Wallis test (non-parametric analog of the one-way ANOVA) to compare the different treatments. *p* < 0.05 was chosen as the threshold for statistical significance.

## Results

### ARPE19 cells cultured on transwell filters display polarized features and different kinase activities

**Figure 1:**
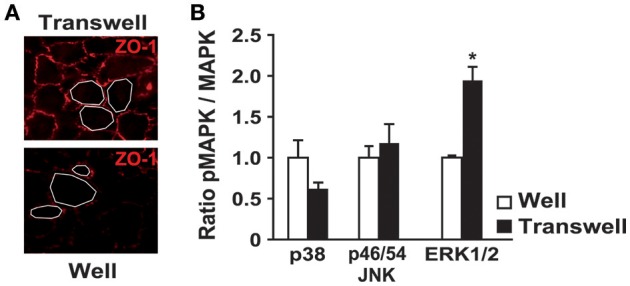
**Polarization of ARPE19 cells by culturing them on transwells modifies cell morphology and basal kinase activities**. ARPE19 cells were cultured either on plastic or on laminin-coated transwell filters as described in material and methods. **(A)** ZO-1 immunofluorescence on ARPE19 cells cultured on both distinct conditions. **(B)** Quantification of basal MAPKs activation by western blot analysis of the phosphorylated and the non-phosphorylated p38, JNK p46/p54 and ERK1/2 after culture in both conditions. Results are expressed as mean ± SEM of 5 experiments with 100% fixed for the culture on well; ^*^p < 0.0012.

We first compared MAPK activities between ARPE19 cells cultured on plastic and on laminin-coated transwell filters. ARPE19 cells cultured on transwell filters showed polarized features as shown by the expression of tight junction zonula occludens (ZO-1) and by the homogeneity of cell size (Figure 1A). Moreover, transepithelial resistance (TER) was similar to that previously described by Dunn et al. (1996 data not shown). As described in a previous study, gene expression is very different when ARPE19 cells are polarized (Turowski et al., 2004). Therefore, we analyzed the activities of the three major kinases, including p38, JNKp46/54 and ERK1/2, in both conditions. Interestingly, no significant difference was observed for p38, and JNKp46/54 activities, whereas ERK1/2 activity is increased 2-fold when ARPE19 cells are cultured on transwell filters (Figure 1B).

### Short- and long-term effects of bis-retinoid A2E on MAPK activities

**Figure 2:**
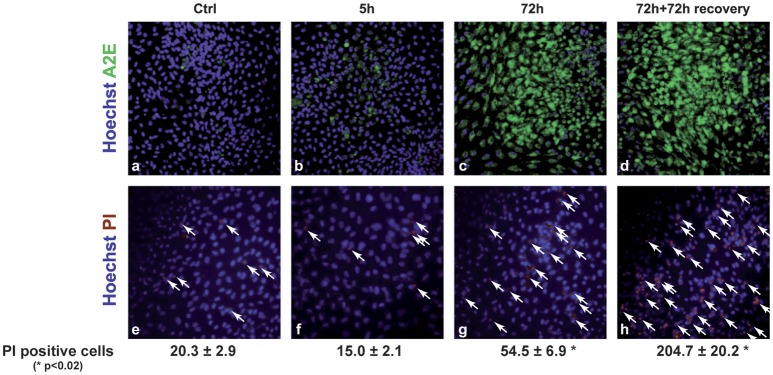
**A2E induces cell death in polarized ARPE19 cells**. Autofluorescence (excitation at 488 nm and emission at 515–540 nm) of A2E was used to show the molecule accumulation into cells. Different periods of time were tested, in details 5, 72, and 72 h followed by 72 h of recovery. HOECHST/PI assay was done for each condition in order to show the cell death induced by A2E. Results are representative of 3 experiments.

**Figure 3:**
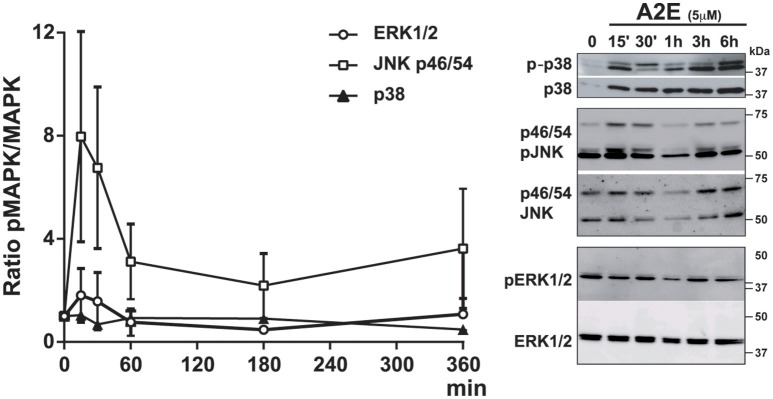
**A2E induces a rapid activation of JNKp46/p54 phosphorylation in polarized ARPE19 cells**. ARPE19 cells were cultured as described in material and methods before treatment with 5 μM A2E for different periods of time. Quantifications of MAPK activation by Western Blot analysis of the phosphorylated and the non-phosphorylated p38, JNK p46/p54, and ERK1/2 after different times of A2E incubation. Results were representative of 3–4 experiments (*n* = 3–4).

**Figure 4:**
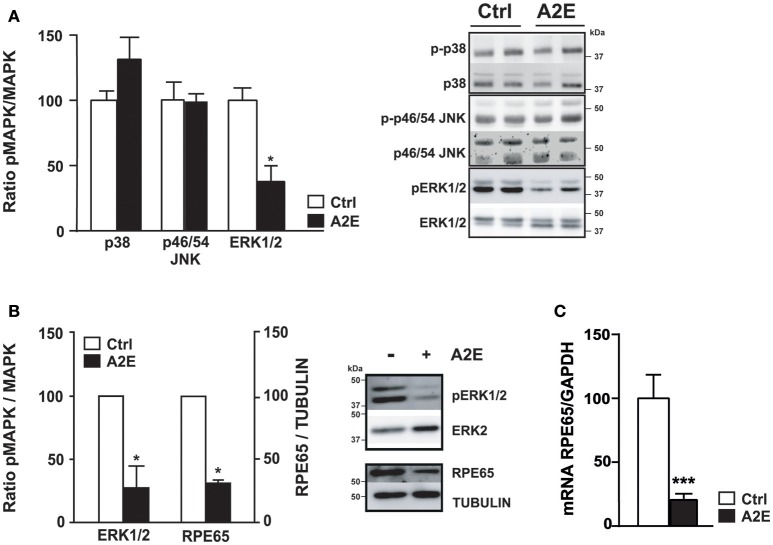
**A2E decreases the activity of ERK1/2 in polarized ARPE19 and isolated mouse RPE cells, and decreases RPE65 protein expression via mRNA down-regulation**. ARPE19 and isolated mouse RPE cells were cultured as described in material and methods before treatment with 5 μM A2E for 72 h following by 72 h recovery. **(A)** Western blot analysis of polarized ARPE19 cells shows the phosphorylated and non-phosphorylated forms of different MAPK, namely p38, JNK p46/54 and ERK1/2 after A2E treatment (72/72 h recovery). Results are expressed as mean ± SEM of 2–3 experiments (*n* = 6–9) with 100% fixed for the untreated cells; ^*^p < 0.01. **(B)** Western blot analysis of isolated mouse RPE cells shows the decrease of ERK1/2 phosphorylation and the decrease of RPE65 proteins expression. Quantification of ratio pERK1/2/ERK1/2 and RPE65/Tubulin are expressed as mean ± SEM of 3 distinct experiments with 100% fixed for the untreated cells; ^*^p < 0.01 **(C)** Quantitative PCR was done to show the level of *RPE65* mRNA in ARPE19 cells. Quantification of the mRNA *RPE65/GAPDH* ratio is expressed as mean ± SEM of 4 experiments (*n* = 11–13) with 100% fixed for the untreated cells; ^***^p < 0.0001.

We analyzed the effect of A2E compound on polarized ARPE19 cells. As previously shown by Sparrow et al., A2E induces cell death (Sparrow et al., 1999). In our conditions, 5 μM A2E already induces PI-positive cells after 5 h, with a significantly stronger effect after 72 h of exposure (5 h: 15.0 ± 2.1 vs. 72 h: 54.5 ± 6.9, *p* < 0.02). Interestingly, if the medium is changed and cells cultured for another 72 h period, A2E is still present in the cells as shown by A2E auto-fluorescence (excitation at 488 nm and emission at 515–540 nm); in fact in this condition, we observed more cell death as shown by PI-positive cells (Figure 2) (72 + 72 h: 204.7 ± 20.2, *p* < 0.02). We then assessed short- and long-term effects of toxic A2E compound on kinase activities. Figure 3 shows a rapid activation of JNKp46/54 within 15 min, while little or no change was observed for p38 and ERK1/2 activities. On the other hand, when we exposed cells to A2E for a period of 72 h, with a 72 h period of recovery (medium is changed after 72 h), we observed a marked decrease of ERK1/2 activity (more than 50%) while no modification of JNKp46/54 and p38 was observed (Figure 4A). The A2E-induced decrease of ERK1/2 activity was also measured when ARPE19 cells were exposed to the compound during a lesser period of time (24, 48 h) (data not shown) and when A2E was added to non-polarized ARPE19 cells (Supplementary Figure 1A).

A similar decrease of ERK1/2 activity is also observed in isolated mouse RPE cells cultured in transwell filters (Supplementary Figure 1B) in the related conditions (Figure 4B). Interestingly, the expression of RPE65 protein, a marker of RPE cells, is also decreased to about 50% after A2E treatment (Figure 4B). In ARPE19 cells, RPE65 protein is not detectable, in non-polarized or polarized cells, while an important level of mRNA is evident (Maeda et al., 2013 and data not shown). However, when we tested the effect of A2E on RPE65 mRNA expression to analyze a potential transcriptional effect of A2E, we were able to detect a significant decrease due to the toxic compound in polarized ARPE19 cells (Figure 4C).

### A2E drastically increases bFGF MRNA expression through a decrease of ERK1/2 pathway

**Figure 5:**
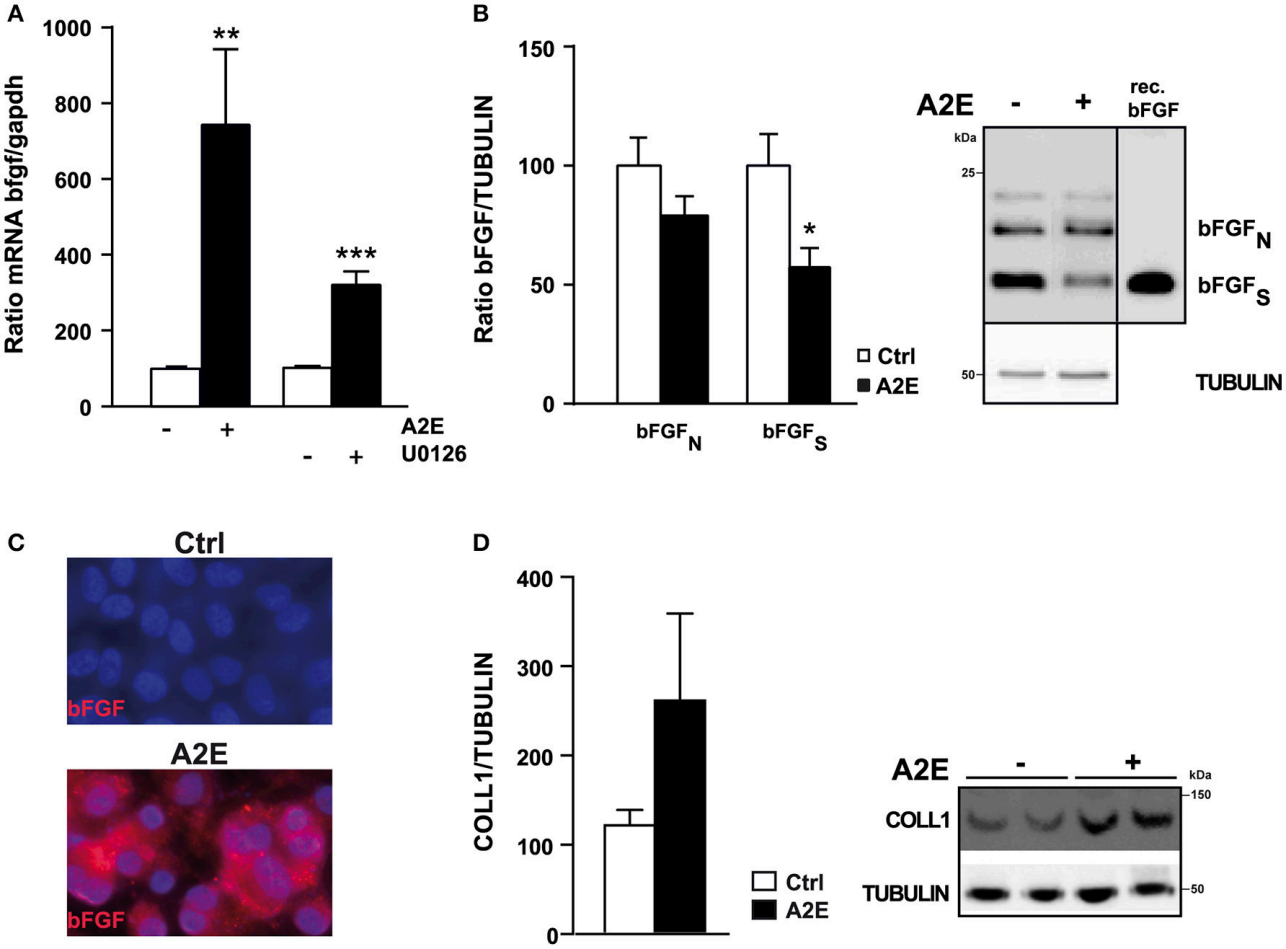
**A2E induces an increase of *bFGF* mRNA at transcriptional level in polarized ARPE19 cells**. ARPE19 were cultured as described in material and methods before treatment with 5 μM A2E for 48 h. **(A)** Quantitative PCR shows the different mRNA levels of *bFGF* in ARPE19 cells treated or not with 5 μM A2E or 5 μM U0126. Quantification of the mRNA *bFGF/GAPDH* ratio is expressed as mean ± SEM of 3 experiments (*n* = 7–14) with 100% fixed for the untreated cells; ^**^p < 0.007, ^***^p < 0.0001. **(B)** Western blot shows the expression level of bFGF (nuclear and secreted forms). Positive control was done using recombinant bFGF [SIGMA, F0291]. Quantification of the bFGF/TUBULIN ratio was expressed as mean ± SEM of 3 experiments (*n* = 9) with 100% fixed for the untreated cells; ^*^p < 0.01. **(C)** Immunostaining of bFGF protein in non-permeabilized polarized ARPE19 cells after 48 h of exposure to 5 μM A2E, result is representative of two distinct experiments. **(D)** Western blot shows the expression level of type 1 COLLAGEN normalized by TUBULIN. Quantification of the COLLAGEN1/TUBULIN ratio was expressed as mean ± SEM of 3 experiments (*n* = 9) with 100% fixed for the untreated cells.

Because of the implication of bFGF in ARMD we were interested in assessing the effect of A2E on this molecule. We observed a strong and significant increase of bFGF mRNA expression after exposure to A2E (7-fold increase). In parallel, the exposure of polarized ARPE19 cells to U0126, an inhibitor of ERK1/2 pathway, led to a significant increase of bFGF mRNA (Figure 5A). Analyses using Cycloheximide and Actinomycin D clearly showed that A2E acts directly at the transcriptional level (Supplementary Figure 2). When we tested the bFGF protein expression, we did not observe any modification; we even found a slight decrease (Figure 5B). This latter result led us to believe that bFGF is secreted from ARPE19 cells. In order to evaluate this hypothesis, we setup an AlphaLisa to detect bFGF protein in the medium of cultured ARPE19 cells (Supplementary Figure 3). The principle of the assay is described in Supplementary Figure 3A with a standard curve using recombinant bFGF illustrated in Supplementary Figure 3B. We were able to measure bFGF at a very low level (50–100 ng) with a good assay linearity up to 3 logarithms. The bFGF protein was detectable by AlphaLisa assay in protein lysates (PS), and in medium samples from cells incubated with A2E (MS), but not in medium samples from control cells incubated with EtOH (Supplementary Figure 3D). Quantification of bFGF protein present in protein lysates (83 ± 7.8 for control and 77 ± 8.0 for A2E treated ARPE19 cells) correlates with Western Blot analysis (Figure 5B), knowing that AlphaLisa did not differentiate between diverse bFGF forms. The very low level of bFGF highlighted a technical problem of detection, which is probably due to the short half- life or to the sticky property of bFGF; to confirm bFGF secretion we assessed bFGF expression by immunostaining the protein in ARPE19 cells treated or not with A2E, but not permeabilized with Triton-X. Figure 5C showed an increase of bFGF at the surface of the cell membrane that confirms the increased trend of bFGF secretion after A2E treatment. In addition, as ERK1/2 has been suggested to modulate type 1 collagen, we tested the effect of A2E on the expression of type 1 collagen protein and interestingly, A2E induces a strong increase of this structural protein (Figure 5D).

### Recombinant bFGF reduces ERK1/2 activity in polarized ARPE19 cells

**Figure 6:**
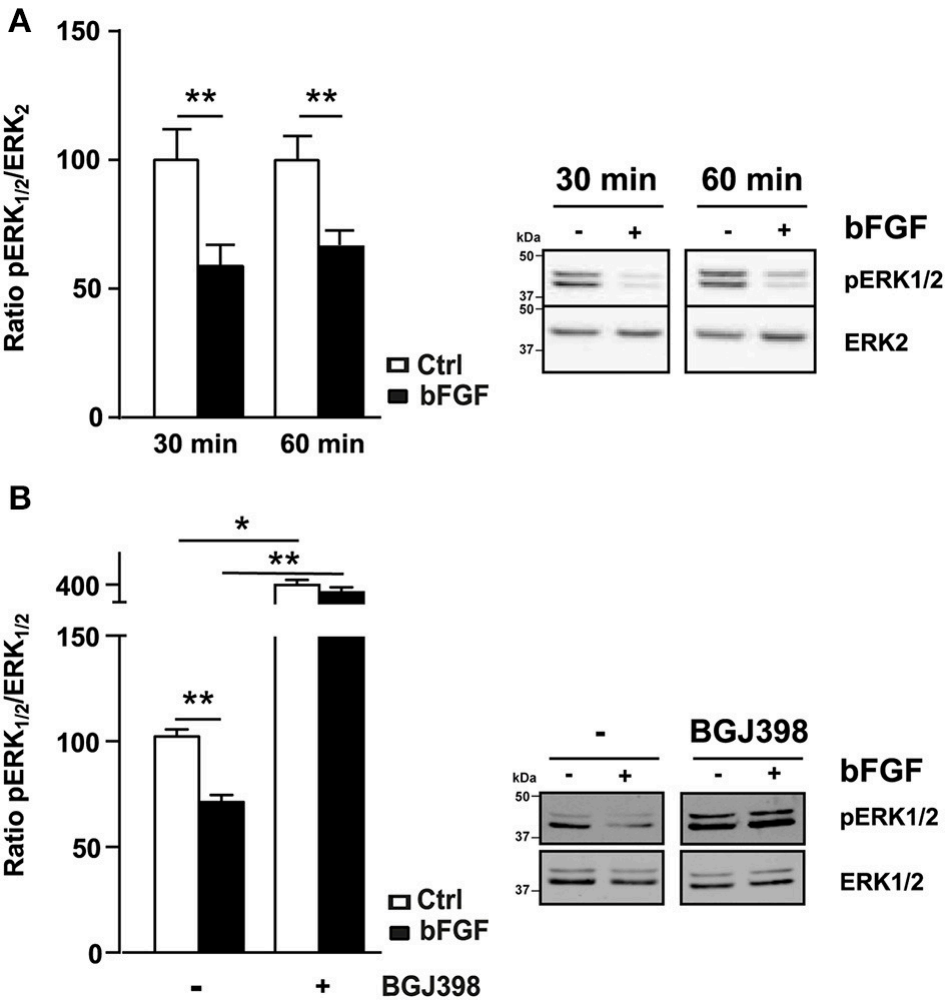
**Impact of bFGF on ERK1/2 activity in polarized ARPE19 cells**. ARPE19 cells were cultured as described in material and methods before treatment with 200 ng/ml of bFGF for 30 respectively 60 min. **(A)** Western blot analysis shows the phosphorylation change of ERK1/2 after bFGF. Quantification of pERK1/2/ERK1/2 ratio is expressed as mean ± SEM of 3-4 experiments (*n* = 9–12) with 100% fixed for the untreated cells; ^**^p < 0.005. **(B)** Inhibition of bFGF receptor, by BGJ398, blocks the bFGF-induced down regulation of ERK1/2 activity, results are expressed as mean ± SEM of 3-4 experiments (*n* = 9–12) with 100% fixed for the untreated cells; ^*^p < 0.001 and ^**^p < 0.0002.

We then tested the effect of recombinant bFGF on ERK1/2 pathway and we observed a rapid decrease of ERK1/2 phosphorylation. After only 30 min bFGF is able to reduce by 50% the activity of ERK1/2 (Figure 6A). When we used BGJ398, an inhibitor of the FGF receptor, we totally blocked the bFGF-induced decrease of ERK1/2 phosphorylation (Figure 6B). Interestingly even in absence of bFGF, but in the presence of the inhibitor, we observed an increase of ERK1/2 activity, probably due to the presence of FGF in the culture medium. In addition, inhibition of the bFGF pathway by BGJ398 did not modify A2E effect on ERK1/2 activity (Supplementary Figure 2B). This result shows that A2E effect on ERK1/2 activity is independent of bFGF, but could be sustained by bFGF.

### A2E and bFGF increase photoreceptor phagocytosis by ARPE19 cells

**Figure 7:**
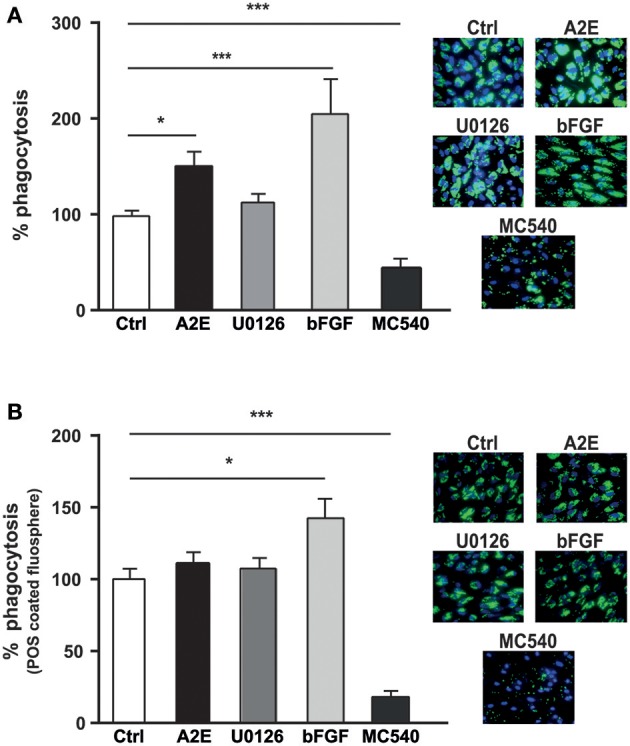
**A2E increases phagocytosis in ARPE19 via bFGF**. ARPE19 cells were cultured as described in material and methods for phagocytosis assay. Merocyanine 540 (MC540), an exogenous photosensitizer able to lower phagocytic activity was used as control and detailed in material and methods. **(A)** Non-specific phagocytosis of synthetic fluorescent beads was performed in absence or presence of 5 μM A2E, 5 μM U0126, 200 ng/ml bFGF or 4.5 μM MC540. Quantification of the ratio between area covered by fluorospheres and the number of cell nuclei is expressed as mean ± SEM of 3–12 experiments (*n* = 9–36) with 100% fixed for the untreated cells; ^*^p < 0.0003 and ^***^p < 0.0001. **(B)** Photoreceptor outer segments (POS) coated with fluorospheres were used to analyze the phagocytic activity of APRE19 in absence or presence of 5 μM A2E, 5 μM U0126, 200 ng/ml bFGF or 4.5 μM MC540. Quantification of the ratio between the area covered by fluorospheres and the number of cell nuclei is expressed as mean ± SEM of 4–7 experiments (*n* = 12–27) with 100% fixed for the untreated cells.; ^*^p < 0.005 and ^***^p < 0.0001.

We tested the effect of A2E on the ARPE19 phagocytosis. Exposure of ARPE19 cells to A2E significantly increased the phagocytosis of fluorescent microspheres, while the ERK1/2 inhibitor did not affect this phagocytic activity. Recombinant bFGF also induces an increase (up to 2-fold) of phagocytosis. As control, we used photodynamic treatment mediated by Merocyanine 540 (MC540) that decreased the phagocytosis of FluoSpheres by 80% (Figure 7A). When the phagocytosis experiment was performed with POS-coated FluoSpheres, we observed an effect only when the cells were exposed to bFGF (50% of increase), while both A2E and ERK1/2 inhibitor did not affect the phagocytic function. In the meantime, MC540, used as control, gave similar results and drastically decreased ARPE19 phagocytosis (Figure 7B).

## Discussion

We first showed in this study that the basal level of MAPK activities is different when ARPE19 cells are polarized. As mentioned, and accepted now by the scientific community working on RPE, the polarization of ARPE19 cells when cultured on filters is essential to preserve the function and gene expression of these cells. We then confirmed previous studies showing the toxic effect of the lipofuscin fluorophore A2E treated (Sparrow et al., 2000) or not (Sparrow et al., 1999) with blue light. Indeed, we used a lower concentration (5 μM) and longer incubation period (72 or 72 h with 72 h of recovery) to completely load polarized ARPE19 cells and induce cell death (Figure 2).

When analyzing kinase activities, we observed a rapid (30 min) and significant activation of the JNK pathway after ARPE19 exposure to A2E, while Westlund et al. did not indicate any activation of this pathway in absence of blue-light photo-oxidation. Besides, cell culture conditions (non-polarized ARPE19 cells), concentration of A2E used (10–100 μM) and time of exposure to the toxic compound were very different from our study and could explain the difference in the results (Westlund et al., 2009). As little A2E is present in the cells after a short-term incubation (Figure 2, 5 h), we could think that the A2E effect on JNK activation is principally due to the triggering of a membrane protein or a receptor expressed at the surface of ARPE19 cells that further analyses would identify. Recently, Fernandes et al. showed that A2E induced secretion of IL-8 pro-inflammatory cytokines via p38 activation (Fernandes et al., 2008), but only when photo-oxidation is induced by blue light treatment. As it was the case in our study, no short-term activation of the p38 pathway was observed in presence of A2E.

Long-term incubation (72 h loading and 72 h recovery) of polarized ARPE19 cells with A2E showed an accumulation of the fluorophore in cells concomitant with cell death (Figure 2); we also observed a significant decrease of ERK1/2 activity which can be reproduced in isolated mouse RPE cells cultured on filters (Figures 4A,B). We previously showed that UVs induce a decrease of the ERK1/2 pathway in ARPE19 cells and we suggested that this could partially explain the UV-induced ARPE19 cell death (Roduit and Schorderet, 2008). Studies have shown that inhibition of the Ras/Raf/MEK/ERK pathway reduces RPE cell proliferation (Hecquet et al., 2002) while other studies have shown that canolol protects ARPE19 cells from oxidative damage probably also through the ERK1/2-mediated pathway (Dong et al., 2011) or that H_2_O_2_ decreases cell viability by activating ERK1/2 (Garg and Chang, 2003). Therefore, the opposing role of ERK1/2 in neurons is difficult to clearly understand because it could be due to the duration of ERK1/2 signaling, the model studied or to associations with other molecular players (Subramaniam and Unsicker, 2010).

Interestingly, we showed that the level of RPE65, a marker of RPE cells, is significantly decreased when isolated mouse RPE cells are exposed to A2E for a long period (Figure 4B). This result together with the fact that A2E drastically decreases RPE65 mRNA expression in polarized ARPE19 cells (Figure 4C), suggests that A2E not only inhibits RPE65 activity by directly binding the isomerohydrolase (Moiseyev et al., 2010), but also by regulating its expression at the transcriptional level. The analysis of the RPE65 promoter suggests a potential AP-1 sequence (Nicoletti et al., 1998) that could explain this result.

Studies by Chaudhary and Avioli have clearly shown in mouse osteoblast-like ME3T3-E1 cells, that ERK1/2 inhibitors (PD98059 and U0126) increase the mRNA expression of type 1 collagen, while an ERK1/2 activator (okadaic acid) inhibits its expression (Chaudhary and Avioli, 2000). These results are in accordance with our result showing the increase of type 1 Collagen protein expression after long-term exposure to A2E, which induces a decrease in ERK1/2 activity. In addition, some studies have shown that RPE cells cultured on type 1 Collagen significantly increase the levels of various angiogenic factors, including bFGF and VEGF. In our study, the increase of bFGF, observed after exposure to A2E (Figure 5A), did not result from the increase of type 1 Collagen, which then induced the increase of the angiogenic factor because when we inhibited protein synthesis with cycloheximide, we still detected a significant increase of bFGF mRNA. This latter result and the inhibition of A2E-induced increase of bFGF mRNA by actinomycin D preferentially suggest an inhibitory effect of ERK1/2 on its targets. Results showing an inhibitory effect of bFGF on the ERK1/2 pathway (Figure 6A) were very surprising because numerous studies showed opposite effects. The high concentration of bFGF recombinant (200 ng/ml instead of 20 ng/ml in most studies) may partially explain this discrepancy, as well as experiments performed in non-polarized ARPE19 cells. Moreover, inhibition of bFGF receptor, by BGJ398, clearly abolishes the bFGF effect on ERK1/2 activity (Figure 6B). In addition, the inhibitor alone increases the basal ERK1/2 activity (without bFGF), probably by blocking FGF activators present in the serum. This latter result suggests that bFGF could decrease ERK1/2 activity depending on its concentration and cell culture conditions used. A recent study, performed by Ferguson *et al*. support our results; indeed they showed that deprivation of bFGF from growth media leads to an increase of RPE-specific proteins, including RPE65, that mediate the differentiation of human embryonic stem cells (hESC) into RPE cells (Ferguson et al., 2015).

In order to test the bFGF secretion, we developed a robust AlphaLisa assay with high sensitivity and a dynamic range of three logarithms. The combinations of strong signals (S) and low background (B) result in very high S:B ratios, even at very low bFGF concentrations (Supplementary Figure 3). Despite the strength of the assay, we were able to detect only a small increase of bFGF in the medium of ARPE19 cells exposed to A2E. Previous studies already mentioned the secretion of bFGF by ARPE19 cells (Mousa and Lorelli, 1999); we confirm herein that A2E, via ERK1/2 down-regulation, induces the secretion of bFGF in polarized ARPE19 cells. This angiogenic factor is found at a high concentration in neovascular tissue in ARMD and is up-regulated in laser-induced choroidal neovascularization (CNV) (Rosenthal et al., 2005) and therefore could be an important factor in ARMD.

Lipofuscin, principally composed of A2E, is formed by the condensation of all-trans retinal and phosphatidylethanolamine; its accumulation is due to incomplete digestion of outer segments (OS) by RPE (Wolf, 2003). A2E-loaded RPE-J and d407-RPE cells internalized similar amounts of OS to control RPE cells, but prevented the clearance of OS via an impairment in lipid degradation (Finnemann et al., 2002). Indeed, A2E inhibits phagocytosis via an oxidative process involving mitochondria only when RPE cells are cultured in physiological concentrations of glucose (5.5 mM, instead of 25 mM present in the RPE medium used in most studies; Vives-Bauza et al., 2008). When we tested the effect of A2E on phagocytic RPE function, we observed a small but significant increase of non-specific phagocytosis (FITC-beads), while specific phagocytosis (opsonized POS) is slightly but non-significantly increased (Figure 7). The difference observed in comparison with other studies could be due to the incubation time we used in our protocol (24 h) or could be specific to ARPE19 cells. Interestingly, bFGF is able to increase both, the non-specific and specific phagocytosis. This latter result is in accordance with the study of McLaren et al. showing that bFGF enhanced phagocytosis in RPE cells isolated from normal rats (McLaren and Inana, 1997). As internal control, we used the photodynamic treatment mediated by merocyanine 540, and we were able to reproduce its inhibitory effect on phagocytosis (Olchawa et al., 2010). We hypothesize that A2E induces, via the ERK1/2 pathway, an increase of bFGF mRNA and subsequently a secretion of the protein, which could then have a paracrine effect on phagocytosis depending on its concentration. A2E, as well as U0126, induces an increase in bFGF mRNA when cells are exposed to A2E or to U0126 for 48 h, but fails to induce enough bFGF protein (incubation for 24 h with A2E for phagocytosis experiment) to impact the phagocytic function. Analysis of the bFGF level in mouse models with high A2E levels (ABCA4^−/−^) or in the retina of ARMD patients will be interesting in order to confirm this hypothesis. This will open a new therapeutic field to treat ARMD.

## Author contributions

RR: conception and design of the work; interpretation of data; final approval of the version. DB, LB, AP, ME, NN: acquisition, analysis and interpretation of data; final approval of the version.CB and LB: preformed the A2E synthesis; final approval of the version.

## Funding

RR and AP were supported by the Gelbert Foundation; RR and LB were supported by the Fritz-Tobler Foundation. AP was supported by the foundation “Art & Vie”.

### Conflict of interest statement

The authors declare that the research was conducted in the absence of any commercial or financial relationships that could be construed as a potential conflict of interest.
