# Supplementary material for: Bis-Retinoid A2E Induces an Increase of Basic Fibroblast Growth Factor via Inhibition of Extracellular Signal-Regulated Kinases 1/2 Pathway in Retinal Pigment Epithelium Cells and Facilitates Phagocytosis
Source: Front Aging Neurosci. 2017 Mar 1;9:43. doi: 10.3389/fnagi.2017.00043 (PMC5331064; doi:10.3389/fnagi.2017.00043)

Supplementary material

Bis-retinoid A2E Induces an Increase of basic Fibroblast Growth Factor via Inhibition of Extracellular Regulated Kinases 1/2 Pathway in Retinal Pigment Epithelium cells.

D. Balmer[1], L Bapst-Wicht[1], A. Pyakurel[1,3], M. Emery[1], N. Nanchen[1], C. G. Bochet[2] and R. Roduit[* 1,3]

*** Correspondence:**
Roduit Raphaël, Ph.D., Jules-Gonin Ophthalmic Hospital, Computational Biology Department, Rue du Bugnon 27, 1011 Lausanne, Telephone: +41 21 692 53 83, FAX: +41 21 626 54 55, Email: raphael.roduit@unil.ch

Supplementary Figure 1. A2E decreases the activity of ERK1/2 in both polarized and non-polarized ARPE19. (A) ARPE 19 were cultured either on transwell filter or on plastic as described in material and methods before the treatment with 5 µM A2E for 72h following by 72h recovery. Western blot analysis of shows the decrease of ERK1/2 phosphorylation in both situations. Quantification of the ratio pERK1/2/ERK1/2 is expressed as mean ± SEM of 3 distinct experiments (n=9) with 100% fixed for the untreated cells; *p<0.003. **(B)** Pigmented mouse isolated RPE (mRPE) cultured on transwell and used in experiments with A2E. mRPE integrity and functionality was tested by measuring transepithelial resistance (TER), not shown.

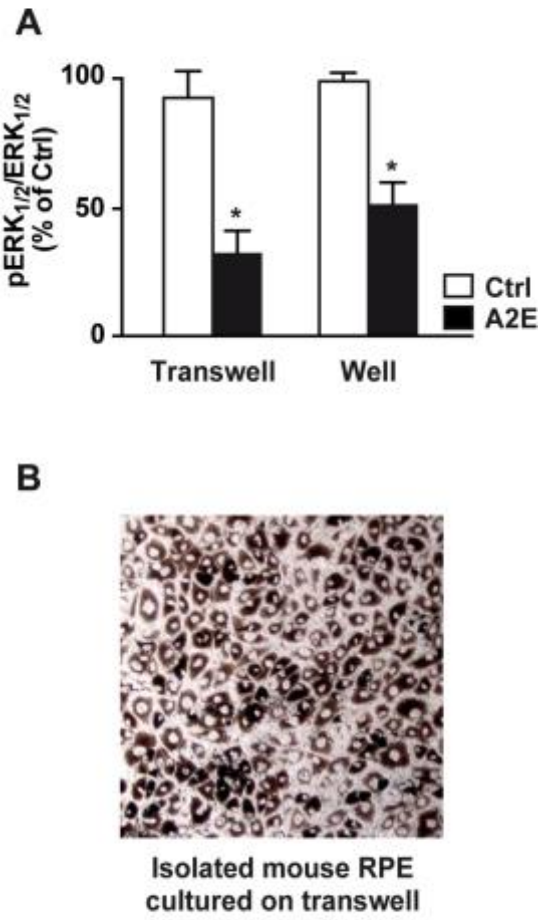

Supplementary Figure 2. A2E induce an increase of bFGF mRNA at transcriptional level in polarized ARPE19 cells. (A) Quantitative PCR of A2E-induced up-regulation of bFGF mRNA in presence or absence of cycloheximide (CycloHex) or Actinomycine D (ActinoD) as described in material and methods. Quantification of the ratio mRNA bFGF/GAPDH is expressed as mean ± SEM of 2-4 experiments (n=6-8) with 100% fixed for the untreated cells; ***p<0.0001 and **p<0.03. **(B)** ARPE 19 were cultured on transwell filter before the treatment with 5 µM A2E for 24h in presence or absence of FGFR inhibitor, BGJ398. Western blot shows that A2E-induced low ERK1/2 activity is independent of bFGF. Quantification of the ratio pERK1/2/ERK1/2 is expressed as mean ± SEM of 2 distinct experiments with 100% fixed for the untreated cells; **p<0.03.

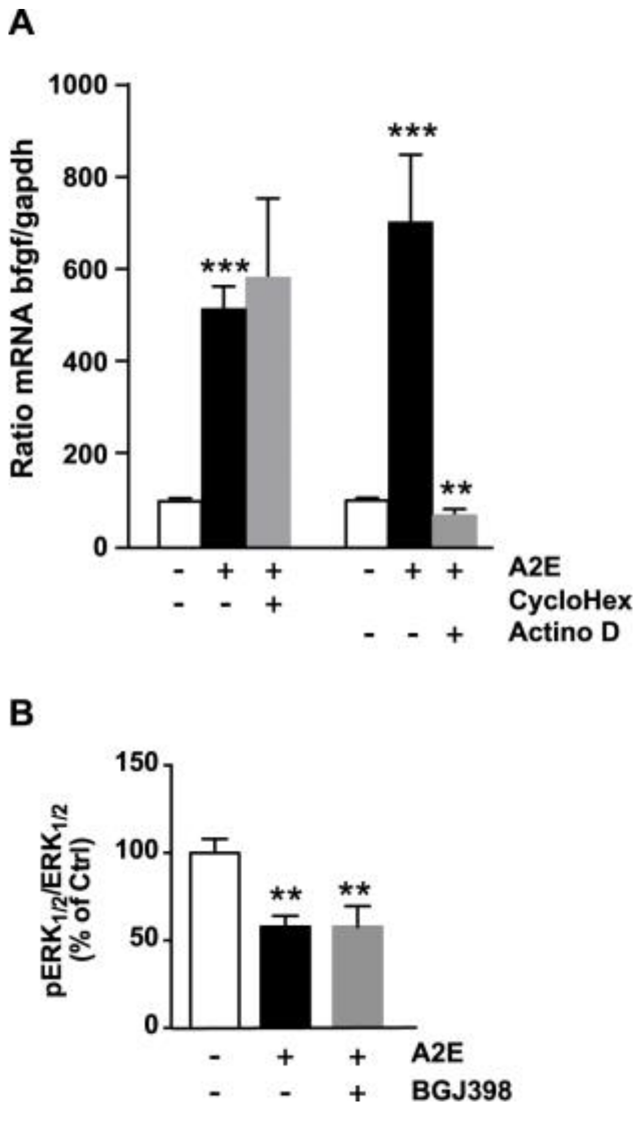

Supplementary Figure 3. AlphaLISA assay to detect bFGF protein. (A) The AlphaLISA bFGF assay is constructed by capturing the bFGF between two specific antibodies, which recognize different epitope of the protein. The protein A–coated donor beads bound one specific antibody, while the other biotinylated specific antibody bound the streptavidin-coated acceptor beads. The presence of all "partners" brings donor and acceptor beads into close proximity (≤ 200 nm). Upon laser excitation at 680 nm, a fluorescent signal is produced and detected between 520 and 620 nm. **(B)** Standard curve of bFGF AlphaLISA assay with an increase amount of recombinant bFGF (10 pg to 30 ng). Five µg of protein lysate (PS) (same sample analyzed by western blot in figure 5C) were used to dose bFGF in polarized ARPE19 cells treated or not with 5µM A2E. Ten fold concentrated medium (MS) resulting of cultured polarized ARPE19 cells in presence or absence of 5µM A2E. **(C)** Quantification of bFGF in protein lysate (PS) and in cultured medium (MS).

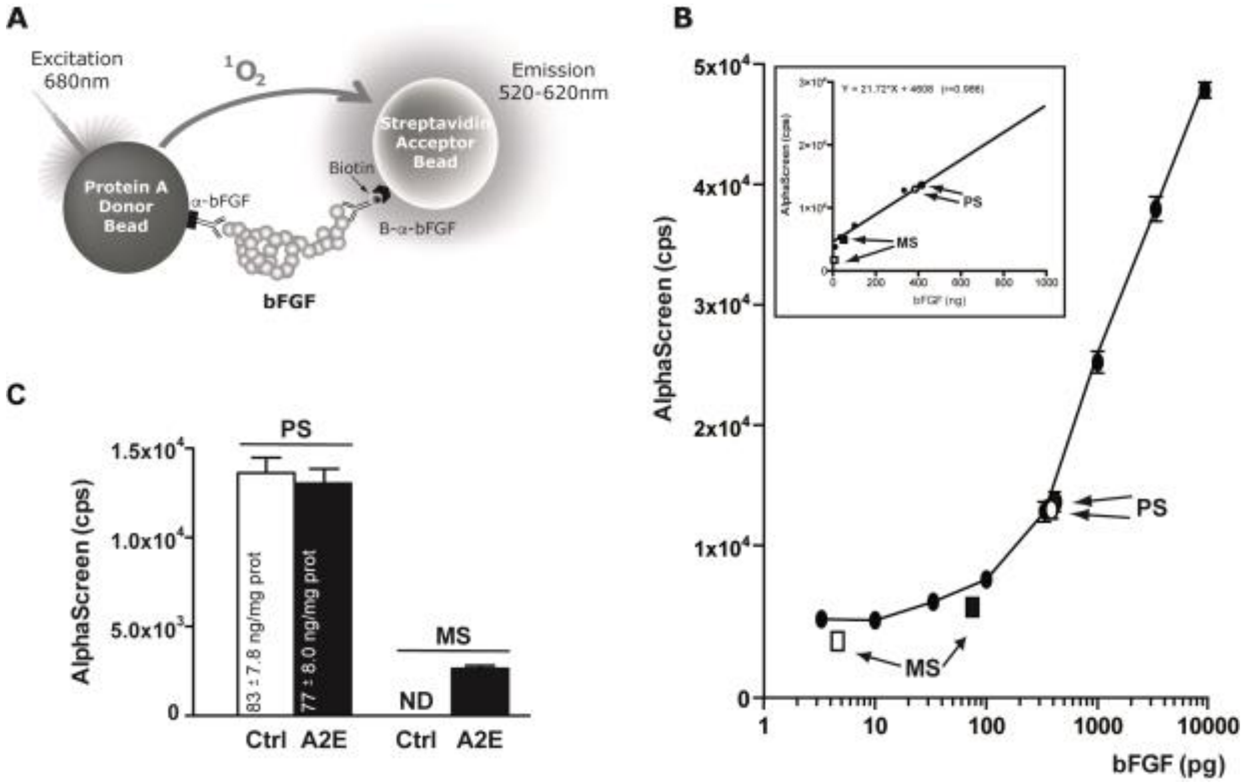

Supplement: Supplementary file 1 [file Image1.PDF]
